# Humic substances mitigate physiological and biochemical responses to waterlogging stress in snake cucumber (*Cucumis melo* var. flexuosus)

**DOI:** 10.3389/fpls.2026.1830185

**Published:** 2026-05-28

**Authors:** Melek Ekinci, Selda Ors, Metin Turan, Murat Aydin, Esma Yigider, Melike Akca, Aslı Cangönül, Ertan Yildirim

**Affiliations:** 1 Department of Horticulture, Faculty of Agriculture, Atatürk University, Erzurum, Türkiye; 2 Department of Agricultural Structures and Irrigation, Faculty of Agriculture, Atatürk University, Erzurum, Türkiye; 3 Department of Agricultural Trade and Management, Faculty of Economy and Administrative Sciences, Yeditepe University, Istanbul, Türkiye; 4 Department of Agricultural Biotechnology, Faculty of Agriculture, Atatürk University, Erzurum, Türkiye; 5 Humintech GmbH Am Pösenberg, Grevenbroich, Germany

**Keywords:** antioxidant enzymes, biostimulants, *Cucumis melo* var. flexuous, fulvic acid, humic acid, nutrient homeostasis, waterlogging stress

## Abstract

**Introduction:**

Extreme rainfall events driven by climate change increasingly cause waterlogging stress in horticultural crops, leading to severe growth inhibition, nutrient imbalances, and oxidative damage. The hypothesis of this study was that humic (HA) and fulvic (FA) acids alleviate waterlogging-induced stress, as reflected in changes in oxidative stress indicators and nutrient dynamics in snake cucumber (*Cucumis melo* var. flexuosus).

**Methods:**

Seedlings were evaluated after 10 days of waterlogging, and HA or FA was applied to the root zone at a rate equivalent to 10 kg ha^-1^.

**Results:**

Waterlogging caused substantial losses in plant growth parameters, chlorophyll content, and macro- and micronutrient concentrations, and increased hydrogen peroxide (H_2_O_2_) levels, malondialdehyde (MDA) concentrations, proline accumulation, and activities of CAT, POD, and SOD. Waterlogging significantly reduced plant growth (up to 36%) and chlorophyll content (up to 52%) and decreased macro- and micronutrient concentrations (up to 80–84%), while increasing hydrogen peroxide (H_2_O_2_), malondialdehyde (MDA), and proline levels, as well as antioxidant enzyme activities. These effects were ameliorated by the application of HA and FA, as evidenced by increases in shoot and root biomass, chlorophyll content, and leaf nutrient uptake under stress conditions. In addition, greater reductions in H_2_O_2_ and MDA levels, associated with lower lipid peroxidation, were observed in the presence of humic substances, along with alterations in CAT, POD, and SOD activities, indicating improved redox homeostasis. Both treatments promoted shoot and root biomass, increased chlorophyll content, and reestablished nutrient homeostasis under waterlogging stress. The regulation of antioxidant enzyme activities and the controlled accumulation of proline suggested improved redox balance between ROS-generating and ROS-scavenging systems in treated plants.

**Discussion:**

The modulation of antioxidant enzyme activities suggests that HA and FA may play a role in maintaining redox homeostasis rather than acting solely as stress-response regulators. In parallel, the increase in macro- and micronutrient concentrations reflects enhanced root function in nutrient acquisition from a hypoxic soil solution. Overall, humic and fulvic acids enhanced waterlogging tolerance in *Cucumis melo* var. flexuosus, highlighting the potential of humic substance-derived biostimulants as a sustainable strategy to improve crop resilience under flooding conditions.

## 1 Introduction

Climate change is increasingly challenging agricultural production worldwide. Abiotic stresses are among the primary factors limiting crop growth, yield, and quality. Although drought and salinity are often considered the predominant climate-related stressors, the increasing frequency and intensity of extreme rainfall and flooding events also pose a serious threat to crop productivity. Flooding occurs when plants or cultivated agricultural areas are partially or completely submerged in water (Kaur et al., 2020; Zhou et al., 2020). Floods affect more than 17 million km^2^ of land every year, damaging plants and causing crop losses, with approximately 10-12% of agricultural areas affected by water accumulation or drainage restrictions (Voesenek and Sasidharan, 2013). Flooding can alter important physicochemical properties of the soil, such as pH, redox potential, and oxygen levels, leaving plants in a stressful environment characterized by oxygen deficiency or absence (Sharma and Kumar, 2023). Under such adverse environmental conditions, plant growth and development are severely restricted, often leading to substantial yield losses and, in extreme cases, plant mortality. Flood stress can lead to oxidative damage caused by the formation of reactive oxygen species (ROS), resulting in responses such as inhibition of stomatal conductance, net CO_2_ assimilation rate, and root hydraulic conductivity in plants. Stress-induced disruption of membrane integrity impairs photosystem efficiency, thereby decreasing the net photosynthetic rate (Ashraf, 2012). Furthermore, flooding impairs plant nutrient uptake, leading to significant nutrient deficiency symptoms (Aslam and Aslam, 2023). In addition, long-term flooding inevitably induces morphological changes, including adventitious root formation and alterations in shoot and leaf development. The inhibition of aerobic respiration caused by flooding limits a wide range of processes, including energy metabolism, seed germination, vegetative growth, and generative development. In this situation, plants attempt to respond by regulating their morphological structures, energy metabolism, endogenous hormone biosynthesis, and signaling processes (Pan et al., 2021).

Various approaches are being used to enhance plant metabolism and increase tolerance to multiple stressors. These practices, which also support plant growth and development, are generally referred to as biostimulants. Biostimulants are substances containing a wide variety of bioactive compounds that generally increase the efficiency of nutrient use in plants and strengthen their tolerance to biotic and abiotic stresses (Ekinci et al., 2015). It has been reported that biostimulants can reduce fertilizer use without affecting yield and quality, stimulate root development, increase the antioxidant potential of plants, enhance leaf pigmentation and plant growth, and promote early flowering (Brown and Saa, 2015; Bulgari et al., 2015). Plant biostimulants that can be used for this purpose include humic and fulvic acids, protein hydrolysates, seaweed, chitosan, silicon, amino acids, various microorganisms, and plant growth-promoting rhizobacteria, as well as similar organic and inorganic substances (Colla and Rouphael, 2015; Yildirim et al., 2021). Humic substances (HS) are high-molecular-weight, heterogeneous, dark-colored, highly dispersed organic compounds that occur naturally in soil, peat, water, and sediments, resulting from the microbial and chemical decomposition of plant and animal organic residues. HS are classified into three groups based on their solubility in alkalis: humic acids (soluble at pH < 2; precipitate at pH < 2), fulvic acids (soluble at all pH values), and humin (insoluble residue) (Zavarzina et al., 2021). HS influence plant responses to abiotic stress by increasing ROS signaling, stimulating proton pump activity, modulating hormonal balance, and activating stress-related genes (Canellas et al., 2024). Humic substances influence plant responses to abiotic stress through multiple physiological processes, including stimulation of root growth, enhancement of nutrient uptake, modulation of antioxidant activity, and regulation of hormonal balance (Canellas and Olivares, 2014; Bulgari et al., 2015; Nardi et al., 2021; Canellas et al., 2024). These compounds have been reported to improve plant performance under stress conditions by promoting root system architecture, increasing nutrient use efficiency, and modulating redox-related processes. Hypoxic conditions not only impair ion transport but also reduce ATP production and promote the overaccumulation of ROS, oxidative damage, and growth inhibition, as recently explored in plant stress physiology. Recent studies indicate that humic substances influence root development, nutrient use efficiency, and antioxidant responses under stress conditions. However, comprehensive studies examining their combined effects on nutrient homeostasis and oxidative stress under waterlogging conditions remain scarce. This paper thus aims to investigate the physiological and biochemical responses of snake cucumber (*Cucumis melo* var. flexuous) to waterlogging stress induced by HA and FA. We hypothesize that the application of humic and fulvic acids is associated with attenuation of the effects of waterlogging stress, as reflected in plant growth, nutritional status, and selected indicators of oxidative damage.

## 2 Materials and methods

### 2.1 Plant material and growth conditions

The experiment was conducted in a pot under controlled greenhouse conditions (25 ± 2 °C during the day, 18 ± 2 °C at night, 50 ± 5% relative humidity). Snake cucumber (*Cucumis melo* var. flexuosus) seeds were initially sown in a peat-perlite mixture (2:1, v:v). Approximately 30 days after sowing, the seedlings were transferred to 1.5-L pots filled with soil:peat:sand (2:1:1, v:v:v). The potting medium had the following characteristics: pH 7.1; EC 161.1 µmhos cm^-1^; lime 3.6%; organic matter 1.9%; NH_4_–N 2.1 ppm; NO_3_–N 1.8 ppm; total N 0.001%; P 2.7 mg kg^-1^; K 220.4 mg kg^-1^; Ca 1738.3 mg kg^-1^; Mg 115.4 mg kg^-1^; Na 12.5 mg kg^-1^; B 0.005 mg kg^-1^; Cu 0.9 mg kg^-1^; Fe 1.8 mg kg^-1^; Zn 0.6 mg kg^-1^; Mn 0.3 mg kg^-1^.

### 2.2 Experimental design and treatments

Before the waterlogging application, Powhumus as humic acid (HA) and Fulvital as fulvic acid (FA) (Humintech GmbH, Am Pösenberg, Grevenbroich, Germany) were applied to the root zone of each potted plant at a rate equivalent to 10 kg ha^-1^ (recommended dose). The humic substances used in this study were derived from leonardite and supplied by Humintech GmbH (Grevenbroich, Germany). The humic acid product (Powhumus^®^) contains approximately 73% organic matter and 10% K_2_O, while the fulvic acid product (Fulvital^®^) contains approximately 33% fulvic acid, 5% Mg, and 80% organic substances. These materials consist of heterogeneous organic fractions characterized by functional groups, including carboxyl and phenolic moieties, that are known to influence nutrient availability and plant physiological responses. The product specifications were based on manufacturer data. Humic acid (HA) and fulvic acid (FA) were applied to the root zone in aqueous solution. The field application rate of 10 kg ha^-1^ was converted to a pot-based equivalent according to the surface area of the pots (15 × 15 cm; 0.0225 m^2^), corresponding to 22.5 mg HA or FA per pot. The compounds were dissolved in water to obtain a final concentration of 0.225 g L^-1^, and 100 mL of solution was applied per plant directly to the soil surface. Pots were placed in large plastic containers filled with water, with the water level maintained approximately 2–3 cm above the soil surface to ensure continuous saturation and hypoxic conditions in the root zone. The water level was monitored daily and maintained for 10 days. The duration of the waterlogging treatment (10 days) was chosen based on previous research indicating that short-term flooding (approximately 7–14 days) is sufficient to trigger hypoxic stress and physiological responses in horticultural crops but is too brief to induce irreversible damage (Barickman et al., 2019). Subsequently, the pots were removed from the water and placed on benches, where the HA and FA treatments continued at one-week intervals. The experiment terminated 50 days after transplanting, at which time morphological, physiological, and biochemical parameters were evaluated.

### 2.3 Growth and morphological measurements

At the end of the experiment, shoot fresh weight, root fresh weight, shoot dry weight, root dry weight, plant height, and stem diameter were recorded. For dry weight determination, plant and root samples were oven-dried at 65 °C for 48 h. Approximately 20 g of fresh leaf samples were rapidly frozen in liquid nitrogen and stored at −80 °C until analysis.

### 2.4 Chlorophyll determination

The concentration of chlorophyll a (Chl a), chlorophyll b (Chl b), and total chlorophyll was determined and expressed as milligrams per gram of fresh weight. Chlorophyll pigments were extracted from fresh leaf tissues using 80% (v/v) acetone, and the absorbance of the extracts was measured spectrophotometrically at 645 and 663 nm following the methods described by Arnon (1949) and Wellburn (1994).

### 2.5 Mineral nutrient analysis

Mineral contents were determined using oven-dried material. Leaf samples were first washed with distilled water, dried at 65-70 °C until constant weight, and then ground. The samples were subjected to wet acid digestion with a nitric-perchloric acid mixture. Nitrogen (N) content was determined using standard Kjedahl procedures (Bremner and Mulvaney, 1982). The other minerals (P, K, Ca, Mg, S, Mn, Fe, Zn, B, Cu, and Na) were analyzed using atomic absorption spectrophotometry (Mertens, 2005a; Mertens, 2005b).

### 2.6 Determination of oxidative stress markers

For H_2_O_2_ analysis, the samples were shaken in a mixer for 30 seconds, and the absorbance was measured at 390 nm using a spectrophotometer. A standard curve was generated using H_2_O_2_ solutions at different concentrations, and the H_2_O_2_ content of the samples was calculated (Shams et al., 2019).

For lipid peroxidation (malondialdehyde, MDA) analysis, the absorbance of the sample from the mixture will be measured at 532 and 600 nm using a spectrophotometer (Shams et al., 2019).

### 2.7 Antioxidant enzyme assays

Fresh leaf samples (0.5 g) were homogenized in 5 mL of ice-cold extraction buffer containing 50 mM phosphate buffer (pH 7.0) and 1 mM EDTA. The homogenate was centrifuged at 12,000 × g for 15 min at 4 °C, and the supernatant was used for enzyme assays.

The method used for determining catalase (CAT) activity is the method applied by Havir and McHale (1987), based on Luck’s (1965) method. CAT activity was determined by monitoring the decomposition of H_2_O_2_ at 240 nm. The reaction mixture contained 50 mM phosphate buffer (pH 7.0), 10 mM H_2_O_2_, and enzyme extract. The decrease in absorbance was recorded for 1 min, and activity was expressed as eu g^-1^ leaf (Gong et al., 2000).

Peroxidase (POD) activity was measured based on guaiacol oxidation at 470 nm. The reaction mixture included phosphate buffer (pH 6.0), guaiacol, H_2_O_2_, and enzyme extract. The increase in absorbance was monitored over time. POD activity determination is based on monitoring the increase in absorbance caused by the colored compound, which is the product of the reaction where guaiacol and H_2_O_2_ are the substrates, at 470 nm, and the results were expressed as enzyme units per g of leaf (eu g leaf^-1^) (Angelini et al., 1990).

Superoxide dismutase (SOD) activity is determined spectrophotometrically by measuring the inhibition of the photochemical reduction of nitro blue tetrazolium (NBT) (Agarwal and Pandey, 2004; Yordanova et al., 2004). One unit of SOD activity was defined as the amount of enzyme that caused 50% inhibition of NBT reduction at 560 nm, and the values were calculated in units g leaf^-1^.

### 2.8 Proline determination

To determine the proline content, the absorbance of the sample taken from the mixture was measured at 520 nm using a spectrophotometer, following the method described by Bates et al. (1973).

### 2.9 Statistical analysis

There were six treatments: C-control (no HA and FA), FA-Fulvic Acid, HA-humic acid, WS-waterlogging stress, WS+FA, and WS+HA. The experiment followed a factorial randomized plot design (3x2), utilizing a total of 108 plants, with three replications and six plants per replicate. Data were analyzed using analysis of variance in SPSS, and mean differences were assessed using Duncan’s multiple range test. Results are presented as mean ± standard error (SE). Treatments sharing the same letter are not significantly different.

## 3 Results

### 3.1 Effects on plant growth parameters

**Figure 1:**
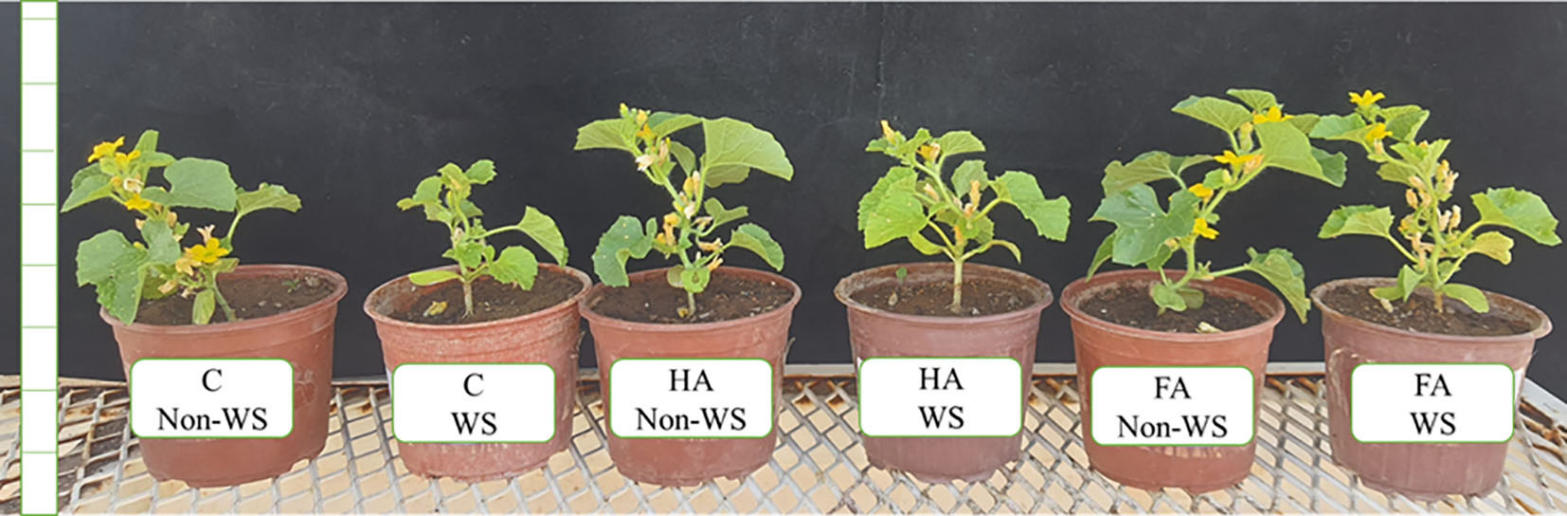
HA and FA effects on the plant growth of snake cucumber seedlings under waterlogging stress. C, control (no HA and FA); HA, humic acid; FA, fulvic acid; WS, waterlogging stress.

While waterlogging adversely affected the growth of snake cucumber seedlings, the HA and FA treatments mitigated its effects (**Figure 1**).

**Figure 2:**
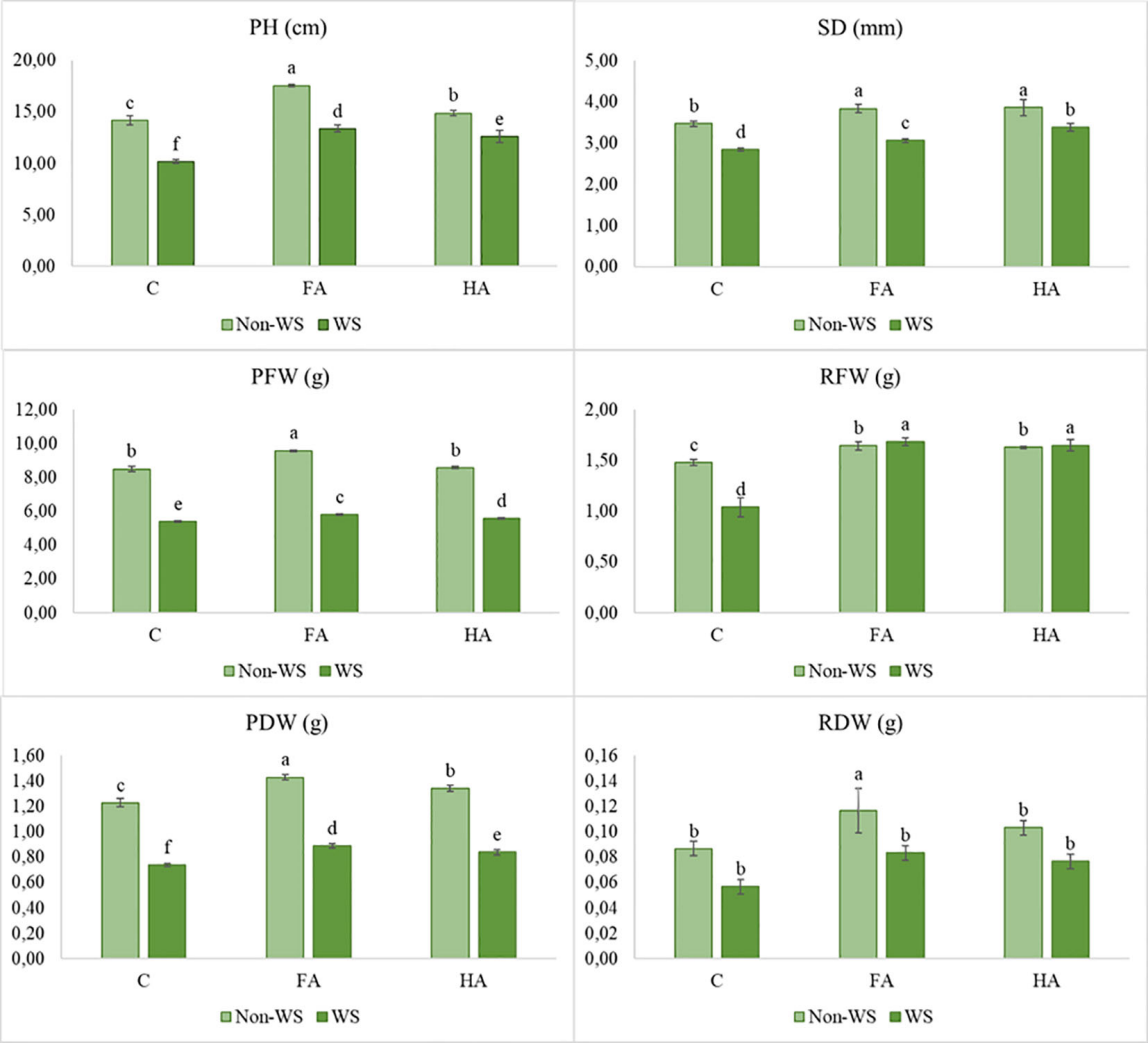
HA and FA effects on PH: plant height (cm), SD: stem diameter (mm), PFW: plant fresh weight (g), RFW: root fresh weight (g), PDW: plant dry weight (g), and RDW: root dry weight (g) of snake cucumber seedlings under waterlogging. Different letters above bars indicate significant differences among treatments according to Duncan’s multiple range test (p ≤ 0.05). The error bars represent standard error (SE). C, control (no HA and FA); HA, humic acid; FA, fulvic acid; WS, waterlogging stress. Light colors: non-stress treatments, Dark colors: waterlogging stress treatments.

The significantly suppressed plant growth due to waterlogging was also indicated by decreases in plant height, stem diameter, and biomass. This decrease, however, was mitigated by the application of HA and FA, resulting in a partial restoration of growth under hypoxic conditions. Waterlogging drastically impaired plant growth as compared to the control. Plant height was reduced from 14.17 ± 0.44 cm to 10.18 ± 0.17 cm (−28%), and shoot growth fresh weight from 8.50 ± 0.17 g to 5.40 ± 0.05 g (−36%). Likewise, root fresh weight decreased from 1.48 ± 0.03 g to 1.04 ± 0.10 g (−30%) under hypoxic conditions, indicating a pronounced suppression of biomass accumulation relative to well-aerated conditions. Humic substances applied in parts compensated for these declines. Shoot fresh weight, identified as one of the important traits for growth under waterlogging, was significantly different compared to stressed control with FA treatment 5.79 ± 0.05 g (+7%), and FA treatment was higher than HA, with +4%. For plant height and root biomass, similar trends were found. Such improvements indicate that humic substances are associated with improved growth performance under stress (**Figure 2**).

### 3.2 Effects on chlorophyll content

**Figure 3:**
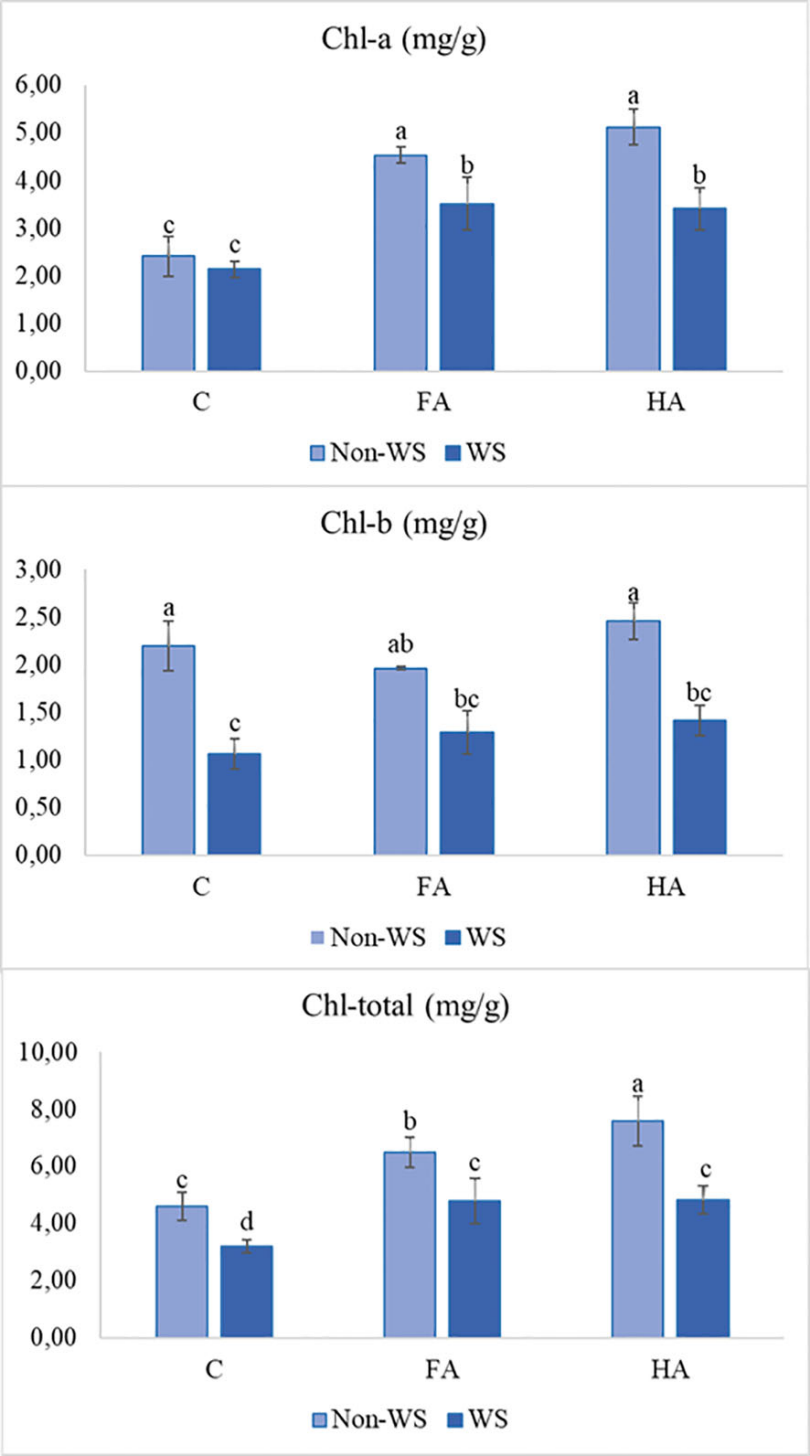
HA and FA effects on Chl-a: chlorophyll a (mg/g), Chl-b: chlorophyll b (mg/g) and Chl-total: total chlorophyll (mg/g) of snake cucumber seedlings under waterlogging. Different letters above bars indicate significant differences among treatments according to Duncan’s multiple range test (p ≤ 0.05). The error bars represent standard error (SE). C, control (no HA and FA); HA, humic acid; FA, fulvic acid; WS, waterlogging stress. Light colors: non-stress treatments, Dark colors: waterlogging stress treatments.

There was a significant reduction in the chlorophyll content due to waterlogging. Chlorophyll a reduced from 2.40 ± 0.42 to 2.13 ± 0.17 mg g^-1^ FW (−11%) and chlorophyll b decreased from 2.20 ± 0.26 to 1.06 ± 0.16 mg g^-1^ FW (−52%); total chlorophyll decreased from 4.60 ± 0.50 to 3.19 ± 0.22 mg g^−1^ FW (−31%). Under stress both HA and FA treatments significantly enhanced chlorophyll contents. Total chlorophyll was increased to 4.80 ± 0.79 mg g^-1^ FW with FA (+50% compared to stressed control), and 4.82 ± 0.48 mg g^-1^ FW with HA (+51%). The increase of chlorophyll content shows that humic substances are linked with better photosynthetic capacity during waterlogging conditions (**Figure 3**).

### 3.3 Effects on mineral nutrient composition

**Figure 4:**
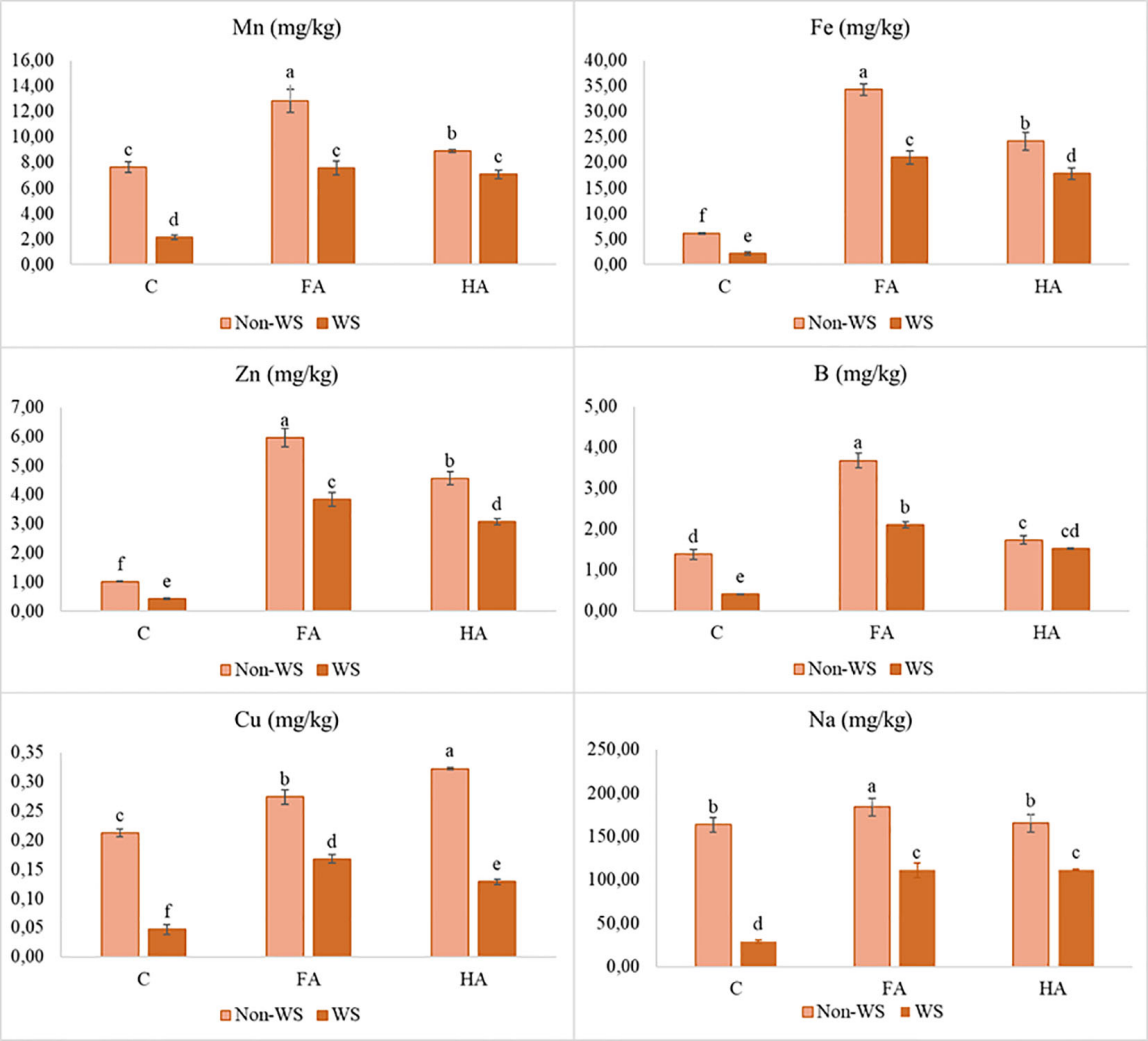
HA and FA effects on Mn (mg/kg), Fe (mg/kg), Zn (mg/kg), B (mg/kg), Cu (mg/kg), and Na (mg/kg) content of snake cucumber seedlings under waterlogging. Different letters above bars indicate significant differences among treatments according to Duncan’s multiple range test (p ≤ 0.05). The error bars represent standard error (SE). C, control (no HA and FA); HA, humic acid; FA, fulvic acid; WS, waterlogging stress. Light colors: non-stress treatments, Dark colors: waterlogging stress treatments.

**Figure 5:**
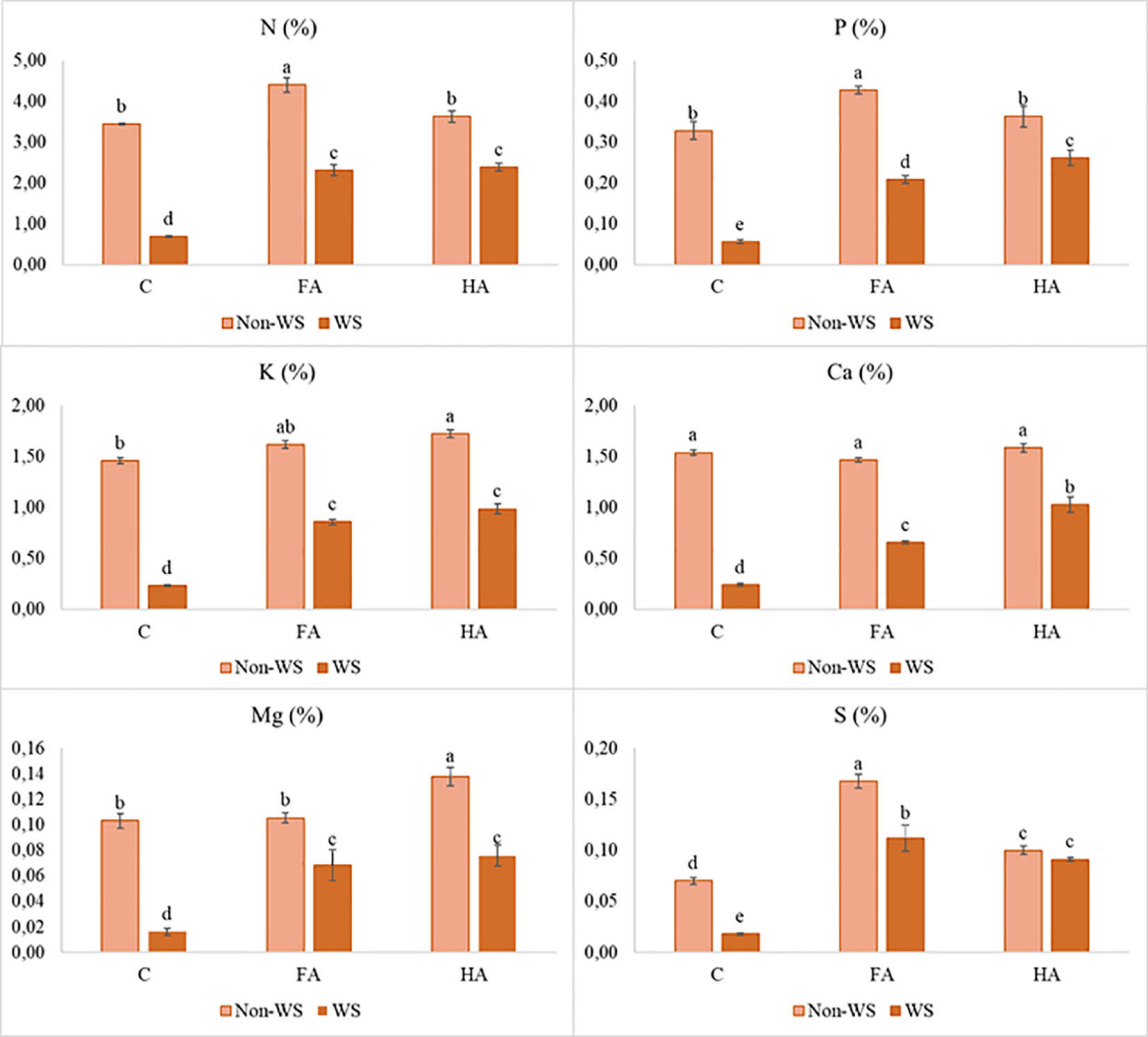
HA and FA effects on N (%), P (%), K (%), Ca (%), Mg (%), and S (%) content of snake cucumber seedlings under waterlogging. Different letters above bars indicate significant differences among treatments according to Duncan’s multiple range test (p ≤ 0.05). The error bars represent standard error (SE). C, control (no HA and FA); HA, humic acid; FA, fulvic acid; WS, waterlogging stress. Light colors: non-stress treatments, Dark colors: waterlogging stress treatments.

Waterlogging severely disrupted nutrient accumulation. Nitrogen content decreased from 3.44 ± 0.02% to 0.69 ± 0.02% (−80%), phosphorus from 0.33 ± 0.02% to 0.06 ± 0.01% (−82%), and potassium from 1.46 ± 0.03%to 0.23 ± 0.01% (−84%). Humic substances were applied at various doses to improve nutrient recovery. HA boosted nitrogen to 2.39 ± 0.09% and phosphorus to 0.26 ± 0.02%, while the increases from FA were more moderate under stress. Similar increases were recorded for Ca and Mg, with S in treated plants. Waterlogging also drastically reduced micronutrient concentrations. Iron content decreased from 6.09 ± 0.27 to 2.16 ± 0.09 mg kg^-1^ (−65%), Zn from 1.02 ± 0.04 to 0.43 ± 0.01mg kg^-1^ (−58%), and Cu from 0.21 ± 0.01 to 0.05 ± 0.01 mg kg^-1^ (−76%). Micronutrient values demonstrated significant improvements for HA and FA applications. FA increased Fe concentration to 21.00 ± 1.32 mg kg^-1^ (+62%) with HA producing 17.84 ± 1.11 mg kg^-1^ (+31%). The results indicate that humic substances help maintain micronutrient availability under stressful conditions (**Figure 4**). These results suggest that humic substances play a key role in maintaining nutrient homeostasis under hypoxic conditions (**Figures 5**, **4**).

### 3.4 Effects on oxidative stress markers

**Figure 6:**
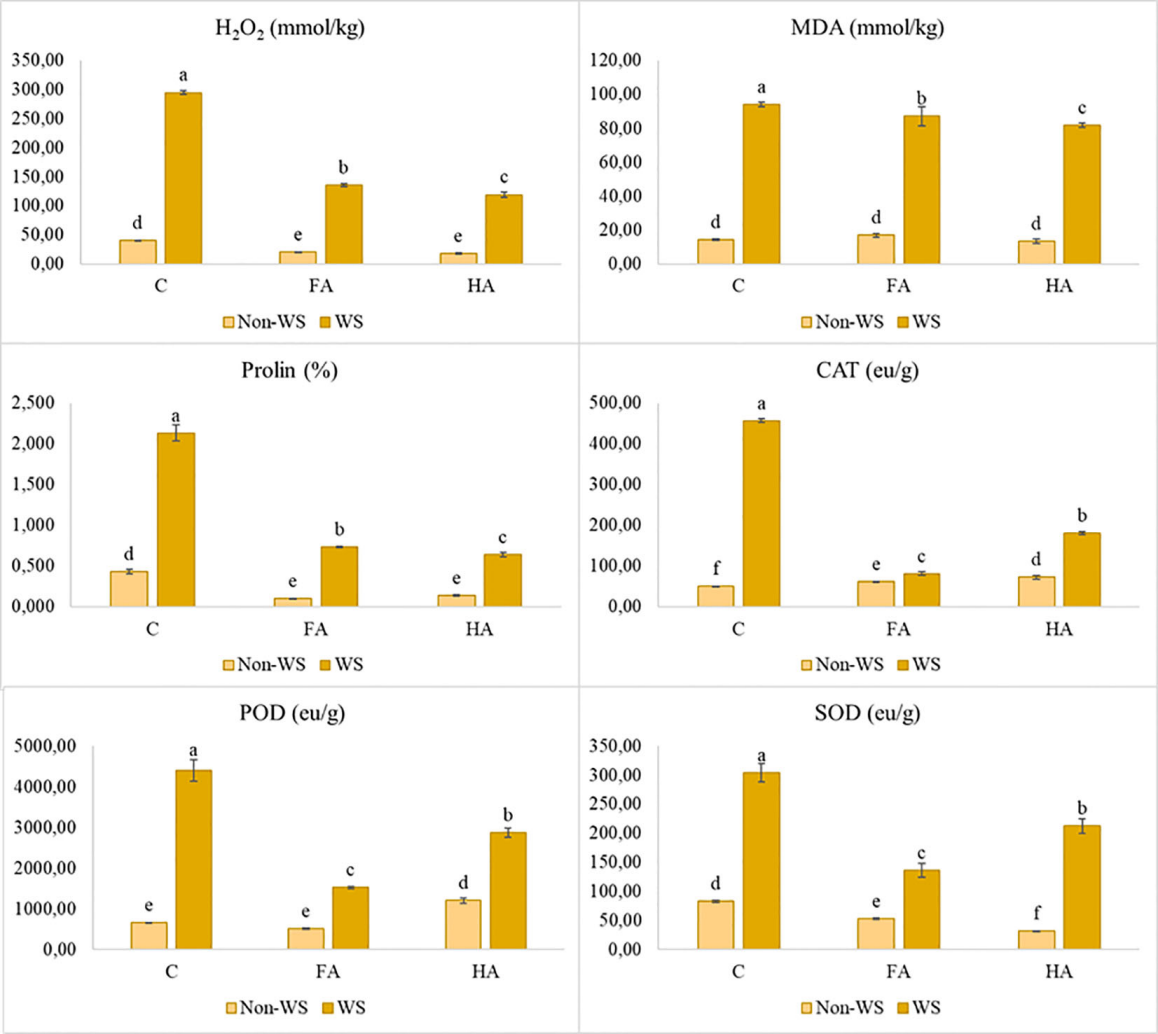
HA and FA effects on hydrogen peroxide (H_2_O_2_, mmol/kg), malondialdehyde (MDA, mmol/kg), and proline (%) contents, catalase (CAT, eu/g), peroxidase (POD, eu/g), and superoxide dismutase (SOD, eu/g) enzyme activity of snake cucumber seedlings under waterlogging. Different letters above bars indicate significant differences among treatments according to Duncan’s multiple range test (p ≤ 0.05). The error bars represent standard error (SE). C, control (no HA and FA); HA, humic acid; FA, fulvic acid; WS, waterlogging stress. Light colors: non-stress treatments, Dark colors: waterlogging stress treatments.

An increase in oxidative stress indicators, such as hydrogen peroxide (H_2_O_2_), malondialdehyde (MDA), and proline content, has been observed under waterlogging stress. An increase suggests improved accumulation of ROS and lipid peroxidation of membrane lipids under stress conditions. Waterlogging stress led to a marked increase in oxidative stress indicators. Compared to the control, H_2_O_2_ levels increased by approximately 1.5–1.8-fold, while MDA levels increased by about 1.6–1.9-fold, indicating enhanced ROS accumulation and lipid peroxidation under hypoxic conditions. Proline content exhibited a stronger response, increasing by approximately 1.8–2.2-fold relative to the control. Compared with stressed plants as the control, the addition of humic substances dramatically decreased the contents of H_2_O_2_ (35%) and MDA (30%). Proline content was further regulated in treated plants. These results suggest that HA and FA reduce oxidative damage by suppressing ROS accumulation and further stabilizing cell membranes (**Figure 6**).

### 3.5 Effects on antioxidant enzyme activities

Waterlogging stress significantly increased the activities of antioxidant enzymes (CAT, POD, and SOD), indicating activation of plant defense mechanisms. Enzyme activity increased roughly about 1.4–2.0-fold under stress conditions compared to the control. On the other hand, the enzyme activities were markedly lower in HA- and FA-treated stressed plants than in untreated plants. This moderated enzymatic response indicates lower antioxidant demand, likely related to reduced oxidative stress in treated plants (**Figure 6**).

## 4 Discussion

Waterlogging stress is a multifaceted limitation on plant growth because it induces a hypoxic microzone in the root zone, leading to alcohol fermentation that displaces aerobic respiration, energy metabolism, and nutrient acquisition (Sakagami et al., 2020). In the current investigation, these limits were explicitly manifested by a simultaneous reduction in plant growth, chlorophyll content, and mineral concentrations, accompanied by a corresponding increase in oxidative stress indicators (**Figures 2**-**4**). These results suggest that waterlogging imposes multiple, interrelated physiological constraints that collectively limit plant performance (Voesenek and Bailey-Serres, 2015; Ma et al., 2022), rather than a single, overly dominant constraint.

The substantial reduction in plant biomass observed under waterlogging conditions coincided with declines in chlorophyll content (**Figure 3**) and nutrient status (**Figures 5**, **4**), suggesting that impaired photosynthetic capacity and nutrient uptake were key contributors to growth inhibition. Chlorophyll decreases, especially for chlorophyll b, could indicate damage to the photosynthetic apparatus or a change in pigment composition that limits light-harvesting efficiency. The high inhibition levels of macro- and micronutrient concentrations recorded in this work reinforce the hypoxic impairment of root function and ion-transport mechanisms. The high inhibition observed for macro- and micronutrient accumulation contents in this work reinforces the notion of hypoxic disruption of root function and ion-transport mechanisms (Kaur et al., 2020; Hasanuzzaman et al., 2017; Pan et al., 2021). Nutrient acquisition is an energy-consuming process, and the limited availability of ATP throughout hypoxia might directly restrict ion-transport systems and nutrient uptake (Kaur et al., 2020). Therefore, the observed decrease in growth likely reflects constrained photosynthetic capacity and impaired nutrient acquisition.

Waterlogging significantly increased oxidative stress indicators, including H_2_O_2_ and MDA, indicating greater ROS production and increased lipid peroxidation (**Figure 6**). These results corroborate earlier reports demonstrating that hypoxic stress induces an oxidative imbalance due to impaired bioenergetics and substrate oxidation (Hasanuzzaman et al., 2017). The higher MDA levels observed in this study also suggest that membrane integrity may have been compromised, thereby reducing cellular compartmentalization and nutrient transport. Moreover, the accumulation of proline under waterlogging conditions is a typical stress response, with proline having been reported to act both as an osmoprotectant and as a marker of the severity of various stress processes (Barickman et al., 2019).

Waterlogging stress markedly increases antioxidant enzyme activities (CAT, POD, and SOD) and elevates H_2_O_2_ and MDA levels, indicating heightened oxidative stress under hypoxic conditions. In contrast, ROS-scavenging enzymes (CAT and APX-ascorbate peroxidase) are induced (Hasanuzzaman et al., 2017), which is reflected in our dataset (**Figure 6**), indicating activation of enzymatic defense systems against excessive accumulation of ROS. The corresponding increase in MDA also indicates extensive ROS buildup, which exceeds the capacity of cellular detoxification mechanisms, thereby causing membrane lipid peroxidation and subsequent perturbation of cellular homeostasis. Oxidative damage is neutralized or scavenged by ROS and their intermediates, which are converted into harmless compounds (Iqbal et al., 2025).

Although waterlogging stress was considerably alleviated by the application of HA and FA, this was reflected in parameters such as biomass accumulation, chlorophyll content, and nutrient content (**Figures 1**-**3**). This increase corresponded to improved chlorophyll (**Figure 2**) and nutrient status (**Figure 3**), indicating that the preservation of photosynthetic capacity- and leaf nutrient availability contributed to better plant performance. The results from these experiments suggest that humic substances may help preserve physiological function in hypoxic environments. Humic substances are known to stimulate root growth, increase phyto-availability of nutrients, and enhance nutrient uptake, probably through their effects on root development and membrane transport processes (Canellas and Olivares, 2014; Atero-Calvo et al., 2024). The improved nutrient status observed in treated plants may have contributed to sustaining metabolic activity under waterlogging conditions. Humic compounds promote plant growth by altering plant root architecture (increased root length, etc.) and shoot growth (branching, etc.) (Nardi et al., 2021). Due to functional groups such as carboxyl, phenolic, hydroxyl, and carbonyl, HA can increase soil cation exchange capacity, improve water retention, and promote the formation and stabilization of soil aggregates. Furthermore, through these effects, as well as by regulating antioxidant enzyme activity and stabilizing cell membranes, HA reduces stress in plants (Nabi et al., 2025). The increase in chlorophyll synthesis and photosynthetic activity caused by humic substances is attributed to their facilitating the uptake and transport of various nutrients within the chlorophyll molecule. Their support of chlorophyll biosynthesis, and consequently their increase in net photosynthesis rate and carbon assimilation capacity, explains their contribution to improved plant growth and performance under stressful conditions (Li et al., 2024; Zhi et al., 2024).

The decrease in H_2_O_2_ and MDA levels in HA- and FA-treated plants also partially supports the hypothesis that humic acids are associated with improved plant cell stability under waterlogging stress. The improved nutrient status, in conjunction with lower ROS levels, may reduce membrane damage relative to the low-N treatment, thereby helping preserve cellular function. The reduction in H_2_O_2_ and MDA concentrations in plants treated with HA or FA that suggests that humic substances may contribute to improved membrane stability under waterlogging stress. Consistent with previous studies, analogous decreases in oxidative damage and increases in membrane stability have also been observed in other plants exposed to abiotic stress following humic substance application (García et al., 2016; da Silva et al., 2023; Atero-Calvo et al., 2024), confirming the consistency of the current results with previous studies. Humic substances have also been reported to be associated with reduced oxidative injury and improved stress tolerance in plants subjected to abiotic stress (García et al., 2016; da Silva et al., 2023), indicating a similar trend. Here, the observed biochemical responses coincided with enhanced growth in the current study and suggest that more productive plants exhibit decreased oxidative damage. While stressors such as H_2_O_2_ and MDA were at very high levels in stressed plants, the increase in stress indicators in plants treated with humic substances was much lower than in control plants, demonstrating that humic substances effectively increase plant tolerance to stress and suppress excessive ROS production under stressful conditions. The presence of functional groups (carboxyl (C (= O) OH) and carbonyl (− C = O) groups containing oxygen (O) attached to the R group) and hydroxyl (-OH) groups in humic substances enables them to regulate ROS accumulation and metabolism (da Silva et al., 2023).

The other component of plant response is reflected in the patterns observed in the activities of antioxidant enzymes (CAT, POD, and SOD). Enzymatic activities responded rapidly and strongly to waterlogging stress, indicating activation of the antioxidant defense system in response to increased ROS levels (Barickman et al., 2019). However, the relatively lower enzyme activities in HA- and FA-treated plants indicate that oxidative stress was reduced, lowering antioxidant requirements. This pattern was consistent with the idea that treated plants had a more balanced redox state, rather than an increased defense response per se. One of the regular plant responses to abiotic stress is proline accumulation (Barickman et al., 2019), but it can have a double-edged effect, depending on the context. High proline levels are commonly used as an indicator of stress severity. On the one hand, proline is an important osmoprotectant that plays a key role in osmotic adjustment, stabilization of cellular structures, protection of proteins and membranes, and scavenging of reactive oxygen species (Aslam and Aslam, 2023). Waterlogging stress is known to significantly increase proline content in plants, as observed in the current study (**Figure 6**). Nonetheless, proline accumulation was significantly lower in HA- and FA-treated plants than in untreated ones, indicating that stress intensity might be alleviated. This indicates that decreased demand for proline accumulation in plants treated with humic substances may correlate with reduced oxidative and metabolic stress, thereby favoring cellular homeostasis. This interpretation is consistent with previous findings that humic substances and related biostimulants modulate proline dynamics and mitigate oxidative stress under abiotic stress (García et al., 2016; da Silva et al., 2023; Atero-Calvo et al., 2024).

Under waterlogging conditions, the application of HA and FA partially restored nutrient ratios. The macro- and micronutrient levels in treated plants were significantly higher than in untreated stressed plants, indicating improved nutrient availability and uptake efficiency (**Figures 4**, **6**). Studies have indicated that humic substances improve nutrient uptake through several mechanisms, including i) root architecture improvements, ii) increasing cation exchange capacity at the rhizosphere, and iii) stimulating membrane transport processes (Canellas and Olivares, 2014; Atero-Calvo et al., 2024). Nutrient availability could play a central role in sustaining metabolic and physiological functions under stress conditions, as improved nutrient status in HA- and FA-treated plants was concomitant with enhanced growth and chlorophyll content. In addition, the simultaneous decrease in oxidative stress markers suggests that increased nutrient status may also help maintain cellular homeostasis by supporting metabolic processes and reducing oxidative damage. Critically, improvements in growth, nutrient status, and markers of oxidative stress occurred concurrently, suggesting that these responses are functionally linked. Nutrient availability is key for cellular metabolism and photosynthesis, whereas excess ROS levels can disrupt membrane integrity and nutrient transport (Kaur et al., 2020; Pais et al., 2022; Hasanuzzaman et al., 2017). Thus, the observed responses may indicate an integrated physiological acclimation, in which improved nutrient status and reduced oxidative injury work together to enhance plant performance under waterlogging conditions. Previous reports have shown comparable integrative relationships between nutrient homeostasis and oxidative stress in plants under abiotic stress (Kaur et al., 2020; Pais et al., 2022). Humic substances have been implicated as potential enhancers of nutrient acquisition by stimulating plasma membrane H^+^-ATPase activity, which drives proton gradients essential for the establishment of ion (ionic balance) transport; and modulating both passive and active ion transport systems, including those involving HKT1-type transporters that play critical roles in maintaining ionic composition under stress conditions (Olaetxea et al., 2019; Khaleda et al., 2017). Further, increased nutrient availability may also be facilitated by improved root growth and rhizosphere interactions. The improvement in nutrient status coincided with increased growth performance and reduced oxidative stress markers, suggesting that nutrient availability may be a key factor in maintaining physiological equilibrium under waterlogging.

## 5 Conclusions

The results of the current study showed that waterlogging stress was associated with extreme changes in plant height, chlorophyll content, nutrient status, and oxidative stress indicators. Consistent effects of humic and fulvic acids applications on positive plant performance [biomass accumulation, partial restoration of concentrations of macro- and micronutrients, as well as lower content of oxidative stress markers (H_2_O_2_ and MDA)] were observed. Crucially, these responses occurred concurrently, indicating an integrated physiological response to conditions of waterlogging. Higher nutrient status may promote metabolic activity and photosynthetic function, while lower oxidative damage may help maintain cellular integrity. However, these associations are based on associative evidence from physiological and biochemical measurements, not direct evidence of regulatory or mechanistic pathways. Given the nature and effects of hypoxia on the plant microenvironment in this specific setting, the broader implications of these results should be interpreted with certain limitations in mind. Additional studies using molecular approaches (e.g., gene expression analysis) will be required to elucidate the specific regulatory networks that drive these responses.

## Data Availability

The raw data supporting the conclusions of this article will be made available by the authors, without undue reservation.
